# Fast preparation of a polydopamine/ceramic composite nanofiltration membrane with excellent permselectivity

**DOI:** 10.1039/d2ra06959h

**Published:** 2022-12-23

**Authors:** Chaoyue Wang, Hanyang Wu, Gaosheng Yu, Haoran Zha, Runlin Han

**Affiliations:** School of Chemistry and Chemical Engineering, Jinggangshan University Ji'an 343009 China hanrunlin@163.com +86-13842712519; Jiangxi Bocent Environmental Technology Co., Ltd Pingxiang 337200 China

## Abstract

Fabrication of a dense polymer/ceramic composite membrane with high permeability remains a great challenge. In this study, a highly selective polydopamine (PDA)/ceramic composite nanofiltration (NF) membrane was prepared by using an Al_2_O_3_ ceramic membrane with pore size of 0.1 μm as the support layer. In order to improve the membrane formation rate, KMnO_4_ was introduced to oxidize the dopamine to improve the reactivity, and Na_2_CO_3_ was used to adjust the pH value of the dopamine solution. When the addition amount of KMnO_4_ is 0.2 g L^−1^ and that of Na_2_CO_3_ is 1 g L^−1^, a functional layer can be formed within 10 min. PDA and polyethyleneimine (PEI) were added to the functional layer to adjust the selectivity of the composite membrane. The composite membrane showed a rejection of 99.7% towards Congo red dye with a high flux of 165 L (m^2^ h bar)^−1^ at ambient temperature. After 3 h treatment with Congo red, the fouling resistance of the membrane was improved compared with that of the ceramic based membrane. The surface morphology and composition of the composite membrane were also characterized with scanning electron microscopy (SEM) and X-ray photoelectron spectroscopy (XPS), which confirmed the successful preparation of the PDA/ceramic composite membrane.

## Abstract 2

Fabrication of a dense polymer/ceramic composite membrane with high permeability remains a great challenge.

## Graphical abstract

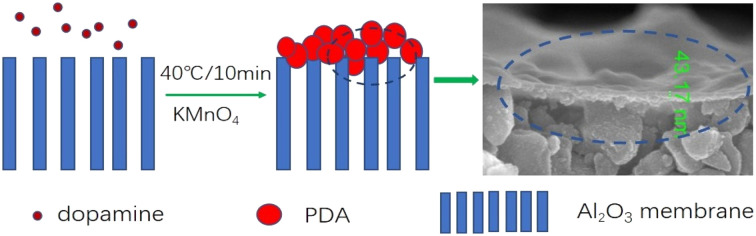

## 1 Introduction

With rapid urbanization, water environment problems are becoming more and more serious. There are many ways to treat waste water, including adsorption, chemical oxidation, precipitation and filtration.^1–3^ However, waste water treatment with low energy consumption is still urgently needed. Membrane separation technology has the advantages of low energy consumption, small footprint and easy integration, and is widely used in the petrochemical industry, biological medicine industry, waste water treatment and other fields. Nanofiltration (NF) with high flux and selectivity is widely used in the precise separation of small molecules during 200–1000 Da and ions with high valence. It is generally believed that the separation mechanism of NF membrane is the synergistic effect of size-sieving and Donnan repulsion.^4^ Most commercial NF membranes are polymeric, because the pore size and surface property of polymer membrane are easy to adjust, while ceramic NF membranes are difficult to obtain because preparation of dense functional layers without defect needs special technology.^5^ The common functional layer materials of commercial composite NF membrane are polyamide and sulfonated polysulfone, while the supporting layer material is polysulfone, polyvinylidene fluoride or polyimide.^6^ However, these kinds of polymer membrane often have some problems, such as low solvent resistance, acid and alkali resistance, oxidation resistance or thermal resistance. In contrast, inorganic membranes such as Al_2_O_3_ membrane, zeolite membrane, β-SiAlON membrane and TiO_2_ membrane, in many cases have high chemical stability, enhanced mechanical strength and high thermal stability, which is of great value in some special separation process.^7,8^

Ceramic ultrafiltration membrane with large pore size and high flux is relatively mature. Nevertheless, low fouling resistance and loose functional layer are still two great challenges to further application of the ceramic membranes. Therefore, an increasing number of studies have been carried out to fabricate ceramic NF membranes with small pore size and hydrophilic surface. In order to change the permselectivity and surface properties of the ceramic membranes, a lot of materials such as hydrophilic polymer, graphene oxide (GO), MXene and nanoparticles have been used to modify the surface of the polymer or ceramic membranes.^9–13^ Chen *et al.* utilized *in situ* chemical deposition method to tune pore size and fabricate TiO_2_ multi-channel nanofiltration membrane with ceramic ultrafiltration substrate. After modification, the average membrane pore radius was changed from 2.4 nm to 0.9 nm.^14^ MnO_2_ nanoparticles were incorporated in the ceramic membranes and the prepared membrane showed high removal efficiency of *p*-chloronitrobenzene.^15^ Li *et al.* used the technique of atomic layer deposition (ALD) to tune the pore size of ceramic membranes. With the increase of ALD cycles, the pore size decreased with a gradient porous structure.^16^ However, the modification technique often needs high energy consumption or complicated preparation process. Polymer coating with high hydrophilicity is common for polymeric composite NF membrane preparation. But polymer/ceramic composite membranes are rarely prepared by depositing polymer on ceramic membranes because of low compatibility between polymer and ceramic materials. There is still great challenge in preparing defect-free polymer/ceramic composite membranes.

Dopamine can spontaneously self-polymerize to form bioinspired polydopamine (PDA) under mild alkaline condition. Recently, PDA has attracted great interest for its wide utilization in surface modification because it can work as a glue on different substrates such as polymer, ceramic and metal.^17–19^ The PDA coating on polysulfone UF membrane can reduce fouling in oily water filtration because it can enhance surface hydrophilicity.^20^ Furthermore, PDA coating has plenty of functional groups which can act as a platform for the post-functionalization. And the excellent hydrophilicity of PDA is meaningful for the antifouling performance of PDA membrane. Mazinani *et al.* used PDA as the coating material to prepare PDA/poly(vinylidene fluoride) NF membrane. The composite membrane showed a pure water flux of 14 L (m^2^ h bar)^−1^ with a MWCO of 550 Da.^21^ Zhao *et al.* reported the fabrication of solvent stable nanofiltration membrane based on an interpenetrating polymer network of dopamine and polybenzimidazole, which needed about 3 days to achieve *in situ* polymerization of PDA.^22^ Recent developments have focused on the challenge of speeding up the deposition time. Alammar *et al.* used PDA as the coating material to prepare NF membrane with high solvent resistance. In order to improve the membrane forming efficiency, 5 mM NaIO_4_ was added and the deposition time can be shorted to 1 h.^23^ Zhang *et al.* used CuSO_4_/H_2_O_2_ and (NH_4_)_2_S_2_O_8_ to accelerate the reaction. The optimized reaction time was 45 min.^24^ Gao *et al.* used PDA coating on the ceramic membrane as a platform to induce the metallization of Ag ions into Ag nanoparticles with CuSO_4_/H_2_O_2_ as a trigger. The PDA coating time was shortened from 16 h to 50 min.^25^. Zhao *et al.* used asymmetric Al_2_O_3_ tubular membranes as supports to prepare PDA/ceramic composite membrane. The formed composite membrane crosslinked with glutaraldehyde showed significant improvement in the rejection of salt solutions and excellent thermal resistance with a flux about 4 L (m^2^ h bar)^−1^. However, the base ceramic membrane with fine pore size of 5 nm was needed in this experiment. Also, it needed 2 h to form the polymer coating and additional crosslinking.^26^ Recently, KMnO_4_ was used as a water-soluble oxidant during PDA coating, which can reduce coating time obviously in a single reaction process and endow glass materials with dense functional layer and durable UV shielding property.^27^ This process route has important inspiration to prepare polymer/ceramic composite membrane efficiently.

Herein, we propose a very easy process to modify the ceramic membrane surface with PDA which is of great significance to promote the industrial application of ceramic NF membrane. The base ceramic membrane was firstly immersed in the PDA solution for several minutes to form skin layer and then dried in oven for 10 min to form dense functional layer. In this design, the KMnO_4_ acts as a trigger to accelerate the polymerization, which decreases the functional layer forming time obviously. The PDA/ceramic composite membrane showed dense and thin skin layer with excellent permselectivity.

## 2 Experimental

### 2.1 Materials and instruments

Flat sheet Al_2_O_3_ ceramic membrane (Jiangxi Bocent Advanced Ceramic, average pore diameter: 0.1 μm) with inner flow channel and dense outer layer has an active membrane area of 842 cm^2^. It was used as supporting membrane to prepare the composite membrane while PDA was used as the functional layer material. Dopamine hydrochloride (AR, Aladdin, shanghai, China) was used as the monomer of PDA. Polyethyleneimine (PEI, MW: 10 000 Da), KMnO_4_, Na_2_CO_3_ and Congo red used in the experiments were all analytical purity grade (AR, Aladdin, shanghai, China) without further purification. The performance of the flat-sheet composite membrane was tested with a dead-end filtration set-up. UV-Vis Spectrometer (752N, Shanghai Youke, shanghai, China) was used to characterize the dye concentration while the conductivity meter (DDS-11A, Leici, shanghai, China) was used to test the salt concentration.

### 2.2 Membrane preparation

In this experiment, PDA functional layer was prepared on the top surface of ceramic membrane by oxidative self-polymerization of dopamine. Firstly, 1 g Na_2_CO_3_ and 0.2 g KMnO_4_ were added into 1 L deionized water with strong stirring, and then 2 g dopamine hydrochloride (DA) was added into the solution and the purple solution turned quickly into black during 20 min stirring. The clean ceramic membrane was immersed into the solution with fixed time to adsorb polymer and form PDA membrane. Then the membrane was taken out and dried at 80 °C for 10 min in oven. At last, the product was cleaned with deionized water for further characterization. For the membrane with PEI, the procedures are all the same except a certain amount of PEI was added in the solution during stirring.

### 2.3 Membrane characterization

The morphology and element composition of the membrane were analyzed by SEM equipped with Energy Dispersive X-ray (EDX) (FEI Nova NanoSEM 450, USA). The samples were sputtered with gold before detection to observe the images of the membranes at the voltage of 15 kV. The elemental composition of membrane surface was analyzed with Al K Alpha Gun with a spot size of 500 μm and 10 scans by XPS (ESCALAB™ 250Xi, ThermoFisher).

The performances including flux (*F*) and rejection (*R*) of the membrane were characterized by a vacuum membrane filtration set-up. In order to decrease the concentration polarization, an aeration system was fixed above the membrane surface. The membranes were tested under 0.1 MPa at room temperature. The concentration of Congo red solution was fixed 0.05 g L^−1^. The permeation flux (*F*) was calculated as follows:1
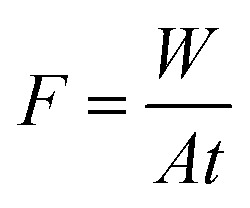
where *W* is the total volume of the water or solution permeated during filtration process; *A* is the valid membrane area; and *t* is the operation time. Rejection, *R*, was calculated using the following equation:2
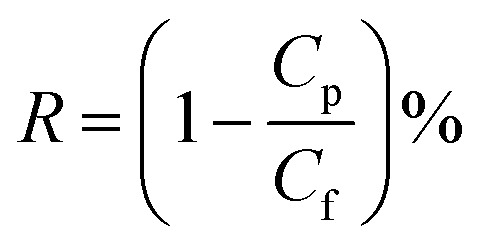
where *C*_p_ and *C*_f_ are the concentrations of the permeate solution and the feed solution, respectively. All the experiments on flux and rejection were tested on three membranes with the average data shown in the paper. For the fouling resistance experiment, the data was tested with one membrane without repetition.

## 3 Results and discussion

### 3.1 Effect of reaction time on the morphology and performance of composite membranes

**Fig. 1:**
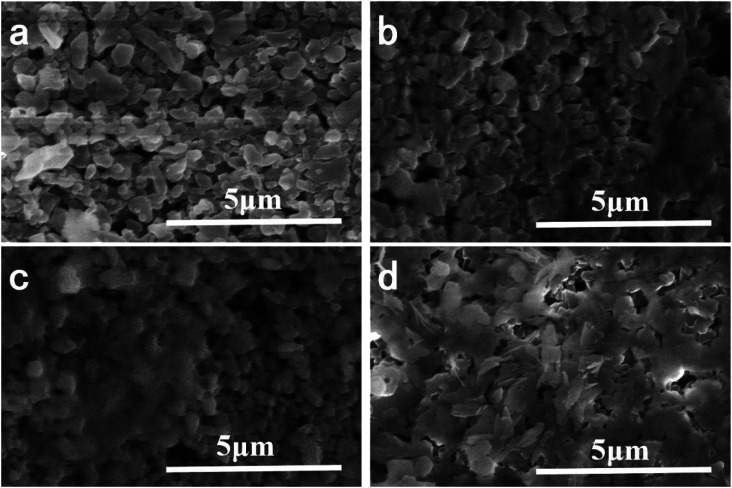
Effect of reaction time on the top surface morphologies of composite membrane ((a) base ceramic membrane; (b) 5 min; (c) 10 min; (d) 15 min).

The morphologies of ceramic membrane and composite membrane were analyzed by SEM with a magnification of 20 000×. The top surface morphologies of the membranes were shown in Fig. 1. The base ceramic membrane showed Al_2_O_3_ particles and large pores in the top surface. When PDA was introduced in the membrane surface at room temperature, part of the membrane was covered by polymer. With the increase of reaction time, denser membrane surface was observed. When the reaction time was increased to 15 min, the surface of the membrane was completely covered by nanoflakes. However, the density of the nanoflakes is not sufficient to plug the pores of the base membrane. So PDA provides a facile and fast method for the surface modification of ceramic membranes with the existence of oxidant at room temperature. The catechol moieties are used to anchor the ceramic membrane and self-polymerization formed highly cross-linked PDA coatings.

**Fig. 2:**
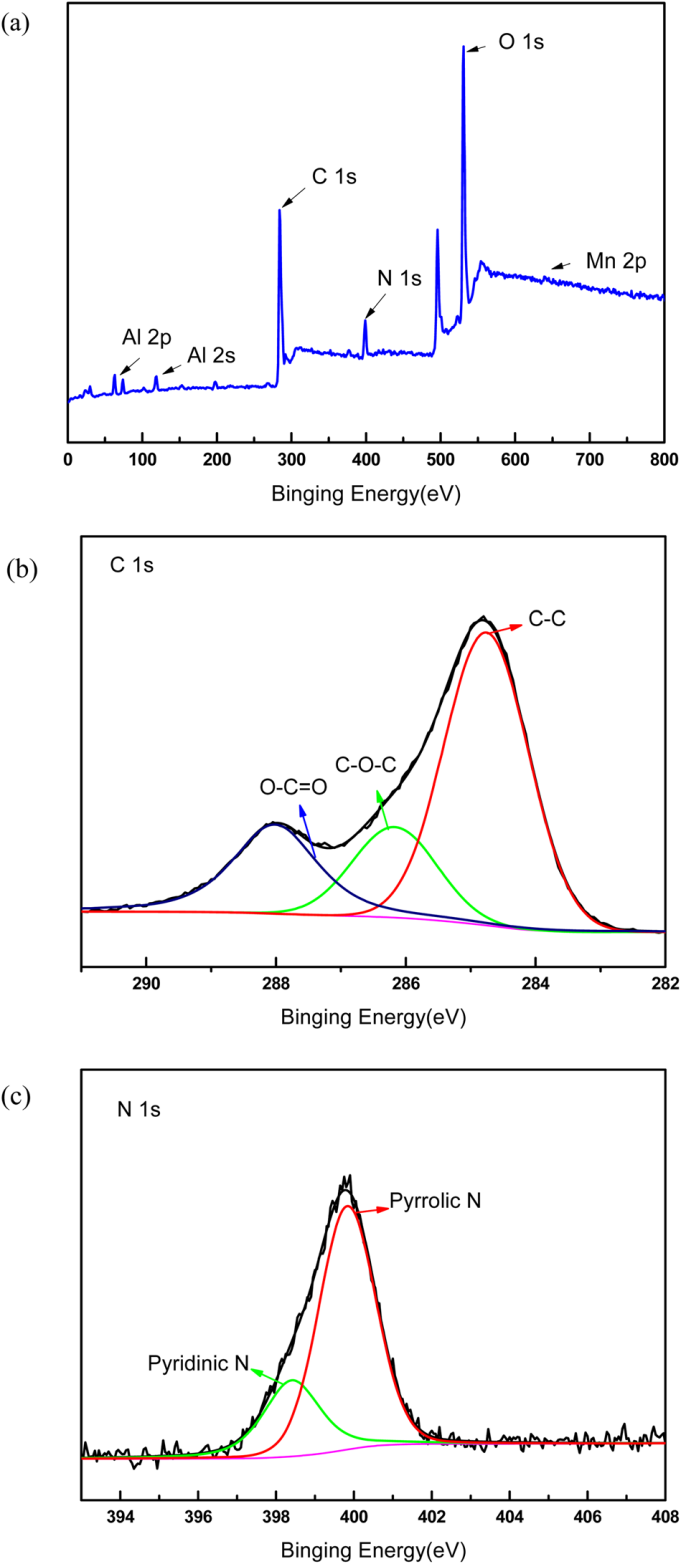
XPS spectra of the composite membrane. (a) Survey spectrum, (b) detailed spectrum of C 1s, (c) detailed spectrum of N 1s.

In order to demonstrate the success of the membrane forming in the top surface, XPS test was performed to verify the success membrane formation on the support membrane. Fig. 2 showed the electron region of the XPS spectra of the membrane surface and high resolution spectra of C 1s and N 1s. The peaks at 284.8 eV and 399 eV were attributable to C 1s and N 1s, which were generated from PDA membrane. The N1s spectrum of the membrane can be fitted into two peaks centered at 398.4 and 399.8 eV, which corresponded to pyridinic- and pyrrolic-like nitrogen, respectively. The small peaks at 74 eV and 120 eV were indexed to Al 2p and Al 2s because the functional layer was very thin and Al_2_O_3_ support membrane was detected. The sharp peak located at 531 eV was attributable to O 1s, which may came from Al_2_O_3_ and PDA. The tiny peak located at 641 eV was attributable to Mn 2p. As part of KMnO_4_ was reduced by dopamine hydrochloride, MnO_*x*_ was formed on the membrane surface, and the peak value of Mn element was very small.

**Fig. 3:**
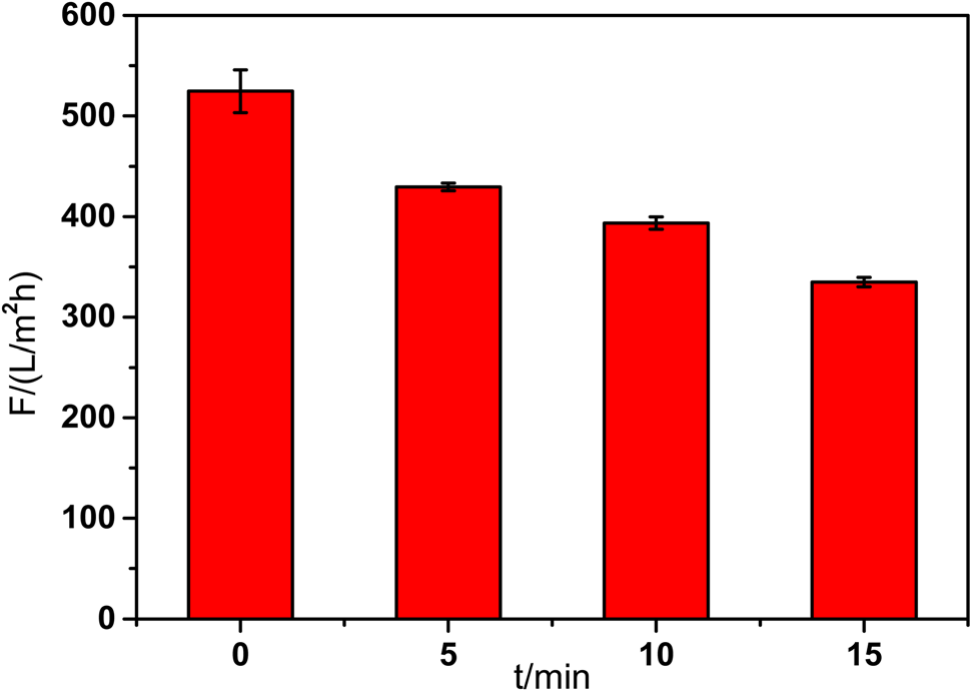
Effect of reaction time on the flux of composite membrane.

The pure water flux of the composite membranes showed in Fig. 3 was tested at 0.1 MPa and room temperature. The base membrane showed the highest flux about 524 L (m^2^ h bar)^−1^. With the increase of reaction time, the membrane flux decreased gradually because of the increase of mass transfer resistance. When the reaction time was 15 min, the membrane flux declined to 335 L m^−2^ h^−1^. The result was in accordance with the SEM images of the membrane top surface. When the reaction time was 10 min, the composite membrane showed reasonable flux and morphology. So the reaction time was fixed at 10 min in the following experiments.

### 3.2 Effect of reaction temperature on the membrane morphology

**Fig. 4:**
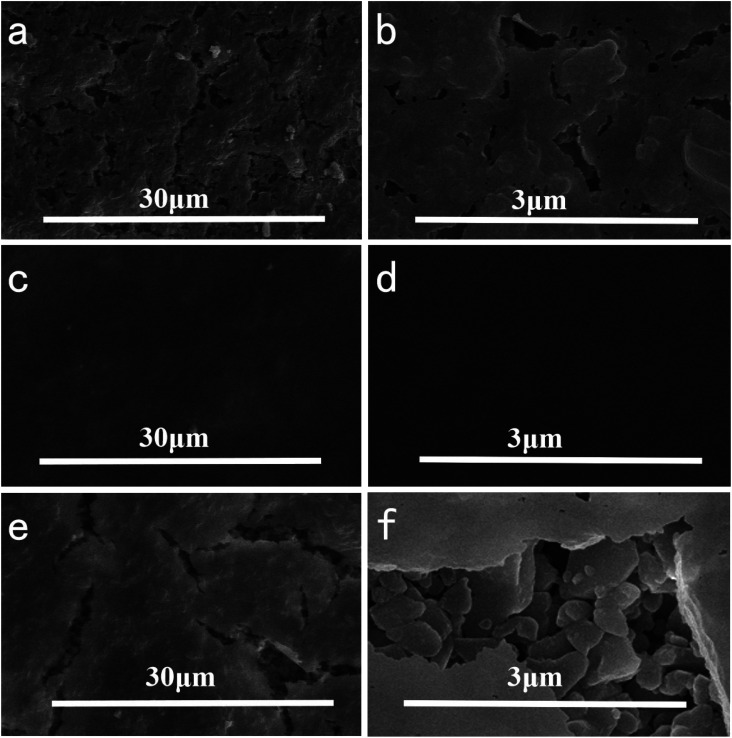
Effect of reaction temperature on the top surface morphologies of composite membrane with different magnifications ((a) and (b) 20 °C; (c) and (d) 40 °C; (e) and (f) 60 °C).

**Fig. 5:**
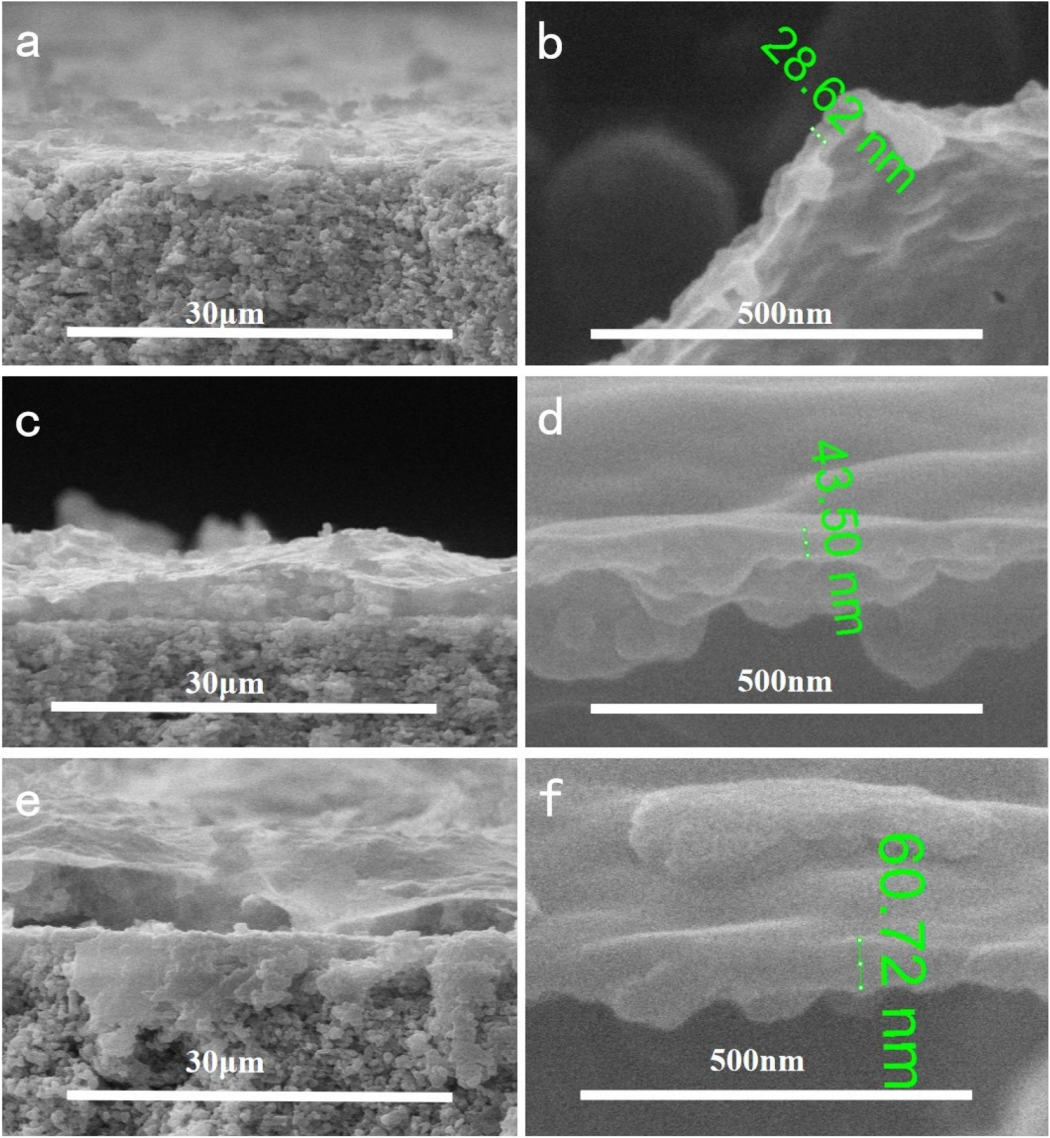
Effect of reaction temperature on the cross-section morphologies of composite membrane with different magnifications ((a) and (b) 20 °C; (c) and (d) 40 °C; (e) and (f) 60 °C).

In order to investigate the effect of reaction temperature on the morphology of the composite membrane, the ceramic substrate membrane was placed in a constant temperature water bath containing 2 g L^−1^ dopamine solution. The results showed that the temperature had a significant effect on the membrane formation. As shown in Fig. 4, the surface of the ceramic membrane was covered by PDA and had some small bumps. At 20 °C, some very small cracks appeared on the upper surface of the composite membrane, which limited the selectivity of the composite membrane. When the reaction temperature was raised to 40 °C, the ceramic membrane was covered with a dense PDA coating. The membrane surface was very smooth without any cracks or pores observed even if the SEM image was magnified 50 000 times. When further heated to 60 °C, long cracks appeared on the surface of the membrane, and the alumina particles of the ceramic base membrane were revealed. The cross-section morphologies of the composite membrane were shown in Fig. 5. It is evident that the dense skin layer of the membrane gradually was thickened from 28.6 nm to 60.7 nm. Higher temperatures led to thicker layers but larger defects, which may be caused by low compatibility and big difference in thermal expansion coefficient between the polymer and ceramic materials.

### 3.3 Effect of PEI content on the membrane morphology and performance

**Fig. 6:**
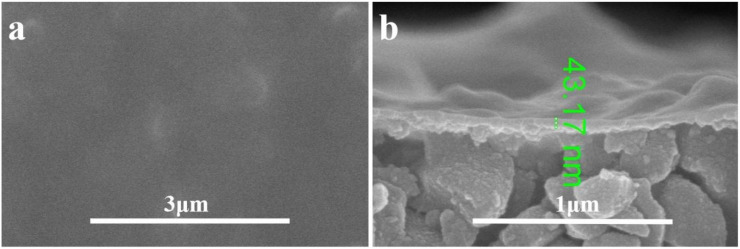
Morphologies of the composite membrane with PEI ((a) top surface; (b) cross-section).

**Fig. 7:**
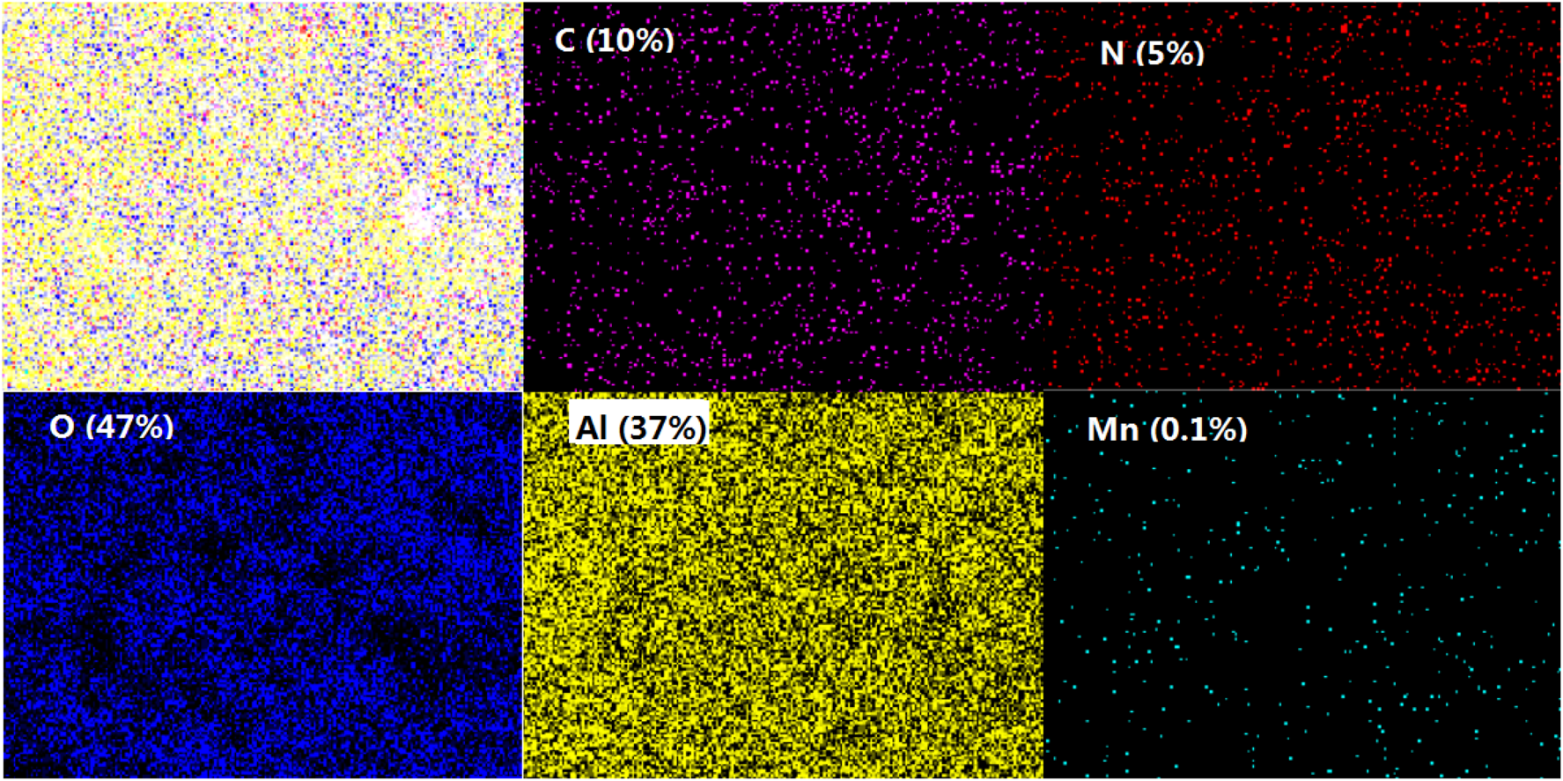
Top surface mapping of the composite membrane with PEI (atomic percentages are shown in the figure).

The composite membrane with 2 g L^−1^ PEI was analyzed by SEM as shown in Fig. 6. The top surface of the composite membrane was very dense without pores detected at the high magnification of 50 000. The ultrathin PDA layer was detected on the Al_2_O_3_ base membrane with a thickness about 43 nm. With the aid of PEI, the membrane without defects was obtained, which is critical to the high rejection of the composite membrane. And the functional layer is ultrathin which is beneficial to the membrane flux. Meanwhile, the elemental mapping images in Fig. 7 showed that N is evenly distributed within the membrane surface with an atomic ratio of 5% which confirmed the effective coating of PDA. The atoms of Al (37%) and O (47%) were observed in the membrane surface because of the Al_2_O_3_ base membrane. Trace element Mn was also found because KMnO_4_ was reduced to Manganese oxide and which was stuck to the surface of the membrane by PDA.

**Fig. 8:**
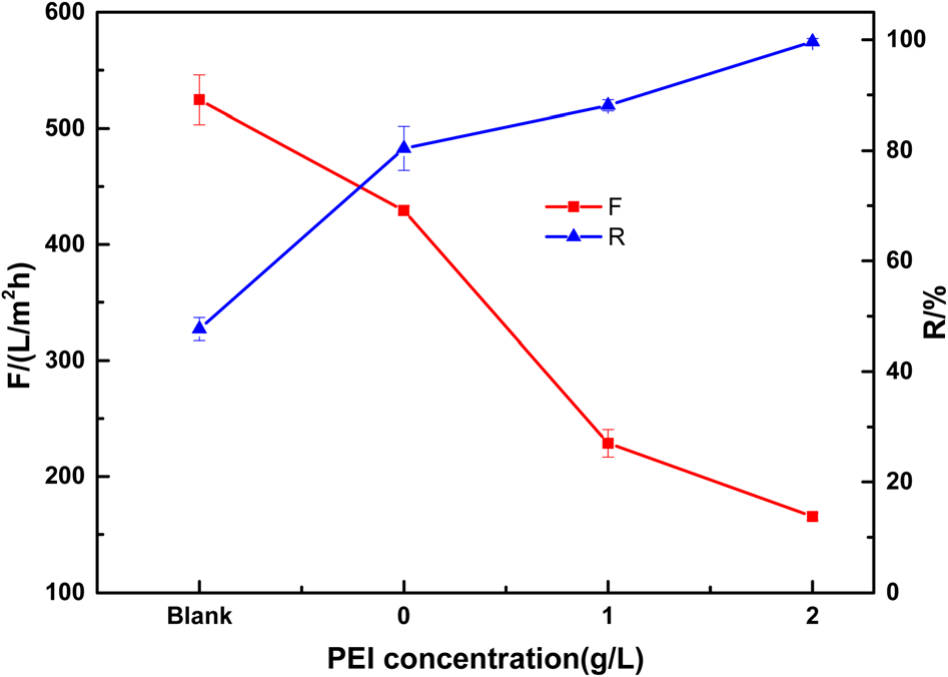
Effect of PEI concentration on the performance of the composite membrane (tested with 50 mg L^−1^ Congo red at 0.1 MPa. Blank, base ceramic membrane).

The effect of PEI content on the performance was studied with different weight ratios of PEI in the PDA solution. As shown in Fig. 8, the blank ceramic membrane has a rejection of 47.7% to Congo red with high water flux of 524 L m^−2^ h^−1^ at 0.1 MPa. When PDA was coated on the ceramic membrane, the dye rejection was improved to 80.4% dye while membrane flux was decreased to 429 L (m^2^ h bar)^−1^. The large pores in the membrane may be blocked by PDA, resulting in a smaller mean pore size which was in accordance with the SEM images. PEI has a significant effect on the membrane properties. When PEI content was increased to 2 g L^−1^, the membrane showed a 99.7% rejection to Congo red with a reasonable flux of 165 L m^−2^ h^−1^. The high dye rejection is due to the very dense layer of the membrane.

As shown in Table 1, PDA coating was time consuming, although the composite membrane showed good permselectivity.^21^ In order to improve the membrane forming rate, oxidants such as CuSO_4_/H_2_O_2_ was used which can accelerate the reaction obviously. The reaction time was shortened to 45 min.^24–26^ The surface modification of ceramic membrane with inorganic particles usually required higher temperature and longer time.^28^ In our work, the modification time was shortened to 10 min and the modification temperature was very low (40 °C), which is very conducive to the production of composite membrane. At the same time, the composite membranes prepared in our work showed high rejection and high flux to Congo red.

**Table 1:** Comparison of the membrane preparation and performance

Membrane	Oxidants	Temperature	Time	Rejection	Permeate	Flux	References
PDA/PVDF	Air	25 °C	24 h	94%	RB5 (991 Da)	14 LMH bar^−1^	21
PDA/PAN	CuSO_4_/H_2_O_2_	60 °C	45 min	90%	PEG 1000	10.1 LMH bar^−1^	24
PDA/Al_2_O_3_	CuSO_4_/H_2_O_2_	25 °C	50 min	—	Oil	1926 LMH bar^−1^	25
PDA/PEI/Al_2_O_3_	—	25 °C	2 h	91%	MgCl_2_	4 LMH bar^−1^	26
TiO_2_/Al_2_O_3_	—	950 °C	2 h	—	Oil	300 LMH bar^−1^	28
PDA/Al_2_O_3_	KMnO_4_	40 °C	10 min	80.4%	Congo red (697 Da)	429 LMH bar^−1^	This work
PDA/PEI/Al_2_O_3_	KMnO_4_	40 °C	10 min	99.7%	Congo red (697 Da)	165 LMH bar^−1^	This work

**Fig. 9:**
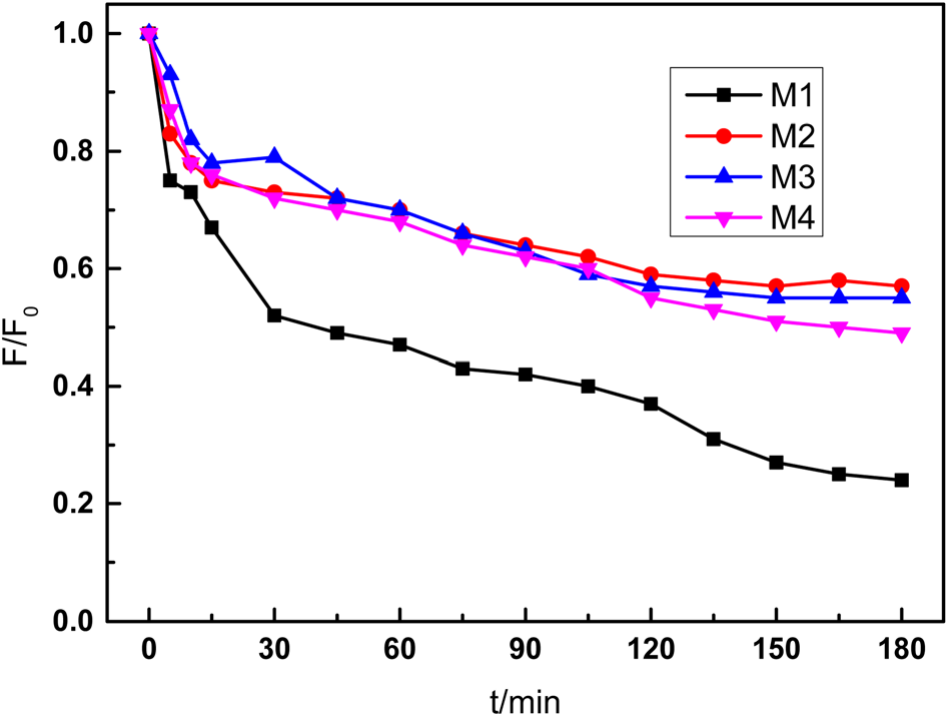
Effect of PEI concentration on the fouling resistance of the composite membrane (tested with 50 mg L^−1^ Congo red solution; M1, blank ceramic membrane; M2, composite membrane prepared with PDA and 1 g L^−1^ PEI; M3, composite membrane prepared with PDA and 2 g L^−1^ PEI; M4, composite membrane prepared with only PDA).

The fouling resistance of the composite membrane was also tested by continuous operation with Congo red solution for 180 min. The control experiment was conducted with base ceramic membrane at the same condition. As shown in Fig. 9, the flux of base membrane decreased quickly during the first 30 min, and the membrane flux decreased to 24% of the initial flux after 180 min operation. When PDA and PEI were introduced in the functional layer, the membrane flux also decreased with extension of operation time because dye molecule also adsorbed into the polymer layer. However, the flux decrease velocity was slower obviously compared with the membrane without modification. After 180 min operation, the flux was decreased to 57% and 55% of initial flux, respectively. For the PDA/ceramic membrane without PEI also showed good fouling resistance because PDA is also hydrophilic material. The membrane flux was declined to the 49% of initial flux after continuous 3 h operation.

## 4 Conclusions

In this study, the PDA/Al_2_O_3_ composite membrane was successfully prepared on the Al_2_O_3_ flat membrane with pore size of 0.1 μm. The composite membrane was prepared with dip-coating method and PDA worked as the functional layer material. KMnO_4_ was introduced to accelerate the rate of polymerization and membrane forming. The membrane preparation time was reduced to 10 min, which significantly improved the preparation efficiency. The functional layer of the composite membrane was very dense and thin, with a thickness of about 43 nm. The results of XPS and EDX mapping confirmed that the PDA functional layer was successfully prepared on the Al_2_O_3_ ceramic membrane, because not only Al and O elements but also N element were observed in the composite membrane surface. The rejection to Congo red of the PDA/ceramic composite membrane was significantly improved from 47.7% to 80.4% while the flux was decreased from 524 to 429 L m^−2^ h^−1^. When 2 g L^−1^ PEI were added in the coating solution, the rejection was increased to 99.7% while the flux decreased to 165 L m^−2^ h^−1^. After continuous operation of 180 min, the Al_2_O_3_ ceramic membrane without modification showed only 24% of initial flux. In contrast, the flux of PDA/ceramic composite membrane with 2 g L^−1^ PEI decreased to 55% of initial flux. The PDA/ceramic composite membrane without PEI also showed 49% of initial flux. The results confirmed the formation of a hydrophilic coating on the surface of the ceramic membrane.

## Data availability statement

The data presented in this study are available on request from the corresponding author.

## Author contributions

Conceptualization, R. H. and H. W.; methodology, R. H.; investigation, C. W. and G. Y.; writing—review and editing, R. H.; all authors have read and agreed to the published version of the manuscript.

## Conflicts of interest

The authors declare no conflict of interest.

## Supplementary Material
